# Mechanical and Dielectric Strength of Laminated Epoxy Dielectric Graded Materials

**DOI:** 10.3390/polym12030622

**Published:** 2020-03-09

**Authors:** Chuang Wang, Qing Sun, Lang Zhao, Jing Jia, Lixiao Yao, Zongren Peng

**Affiliations:** 1 School of Electrical Engineering, Xi’an University of Technology, Xi’an 710048, China; 2 State Key Laboratory of Electrical Insulation and Power Equipment, Xi’an Jiaotong University, Xi’an 710049, China

**Keywords:** epoxy resin, laminated materials, interlaminar interface, mechanical properties, dielectric properties

## Abstract

Laminated epoxy dielectric graded material is a commonly used insulating material with broad application prospects in power equipment. The interlaminar interfaces of laminated epoxy dielectric material between different layers form during its lamination process, and these interfaces are the crucial characteristic structures determining the mechanical and dielectric properties of laminated materials. Therefore, in order to gain a thorough understanding of physic properties behind a certain structural motif, it is necessary to study how these interfacial structures influence the mechanical and dielectric performances of graded materials. In this study, double-layered epoxy resin samples with an interlaminar interface are prepared to study their mechanical and dielectric strength. More importantly, the formation mechanism of the interface, as well as its influence on the mechanical and dielectric strength of this laminated material, is discussed. We found that a cross-linking reaction may take place between epoxy resins at the interlaminar interface, and the degree of cross-linking at the interface should be less than that in the bulk. The mechanical strength of the interlaminar interface is weaker than that of the bulk, and it is reduced by less than 40%. Moreover, the interlaminar interface is inclined to trap carriers, which improves the breakdown strength and arc ablation resistance of the laminated material. Our study of interlaminar interface properties could help in designing epoxy dielectric graded materials with better mechanical and dielectric properties.

## 1. Introduction

Solid insulation materials are widely used in power equipment and electronic packaging as insulating layers and mechanical support [1,2,3]. Epoxy resin is a typical solid dielectric material with excellent processability [4,5,6]. Given the processing and performance requirements, multi-layered epoxy resin materials are used as the insulation structure of power equipment, introducing an interlaminar interface structure between different epoxy resin layers.

Dielectric functionally graded materials (DFGMs) show broad application prospects in the high-voltage and insulation field [7,8]. By optimizing the spatial distribution of dielectric parameters, DFGMs can effectively improve the electric field distribution and the dielectric strength of insulation structures [9,10,11]. There are mainly two methods for preparing dielectric functionally graded materials: centrifugalization and lamination. Both methods can use epoxy resin as the matrix [12,13,14,15]. The method of lamination is easy to industrialize and also shows great potential in three-dimensional (3D) industrial printing [10,11,16]. The graded material obtained via this method has a multi-layered epoxy resin structure in which the interlaminar interfaces are inevitably formed. This interface structure is different from that between two contact samples. The adjacent layers in laminated material near the interface are bonded during the curing process. Therefore, this interfacial structure plays an important role in affecting the mechanical and dielectric properties of the laminated materials.

Similar interfacial structures can also be found in other power equipment. For instance, in some dry-type equipment, multiple pouring and curing processes of epoxy resin are needed, during which interfaces are created in between layers, due to the requirement of a complex structure and large volume [17]. In addition, due to the performance requirements of ultra-high-voltage gas insulation switchgear spacers, a layer of epoxy buffer material is introduced between the conductor and the basin body, resulting in the formation of an interfacial structure between the buffer layer and the epoxy of basin body [18]. Therefore, understanding the properties of this interfacial structure has great significance for designing the insulation material of power equipment.

Despite a vast number of studies concerned with the dielectric and electronic properties of interfacial structures, little is known about the properties of this interlaminar interface in laminated epoxy graded material. The effect of the interface and channel on electric tree growth in epoxy resin was studied, but this interface is created between the two contact layers instead of via lamination in laminated materials [19]. The performance of two kinds of solid–solid interfaces was studied under the pulse voltage, but the electrodes were arranged inside the interface [20]. Other studies on the interlaminar interface of solid insulating materials mainly focused on the space charge characterizations [21,22,23]. The formation and the decay of space charge at different material interfaces were studied, and it was found that the interface plays a role in trapping the electrons [21]. In double-layered epoxy resin, the interface can accumulate negative polar charges under a direct current (DC) field [23]. However, these studies only focused on space charges in a DC field, whereas the dielectric properties of these interfacial structures in an alternating current (AC) field remain to be investigated. In addition, the dielectric properties of multi-layered insulating materials cut from DC cables were also studied [24,25,26,27]. These studies mainly analyzed the space charge characteristics of the interface between cable materials and other polymers at different temperatures and DC electric fields. It was found that the interface space charge consists of a polarization charge caused by interfacial polarization (Maxwell–Wager–Sillars polarization) and a cathode injection charge [27]. All of these studies mentioned above did not cover the mechanical and AC dielectric strength of laminated epoxy graded material.

Therefore, we extend our study to laminated epoxy graded material in order to understand how such interfaces influence the mechanical and dielectric strength of the insulating material. In what follows, we present our research results on double-layered epoxy resin samples. These samples were prepared via two-step pouring and curing processes using the same epoxy formula, which guaranteed that the epoxy material on both sides of the interface followed the same formulation and curing process. By analyzing the mechanical and electrical strength of the samples with and without this kind of interface as a comparison, the influence of this kind of interface on the relative properties was studied.

## 2. Sample Preparation and Experimental Methodology

### 2.1. Materials

In this study, the epoxy resin was the diglycidyl ether of bisphenol A (DGEBP A) with an epoxide value of 3.9 mmol/g produced by Nantong Xingchen Synthetic Material Co., Ltd. (Nantong, China). The curing agent was methylhexahydrophthalic anhydride (MeHHPA) produced by Puyang Huicheng Electronic Materials Co., Ltd. (Puyang, China). The accelerant agent was 2-ethyl-4-methylimidazole produced by Shenzhen Jintenglong industrial Co., Ltd. (Shenzhen, China). All reagents were directly used without treatment.

### 2.2. Instruments and Characterization

An electronic balance (JH1102, China Shanghai Balance Instrument Factory) was used for component weighting. A program-controlled vacuum oven (DZF-2B, China Shanghai Keheng Factory) was used for the curing process of epoxy resin.

The samples were immersed in liquid nitrogen for two minutes and then broken by a clamp into smaller pieces with cut surfaces. Those sample pieces were coated with gold by ion sputtering, and then a Keyence VE-9800S scanning electron microscope (SEM) was used to observe the fracture section, in which the electron gun voltage was set to 20 kV.

**Figure 1:**
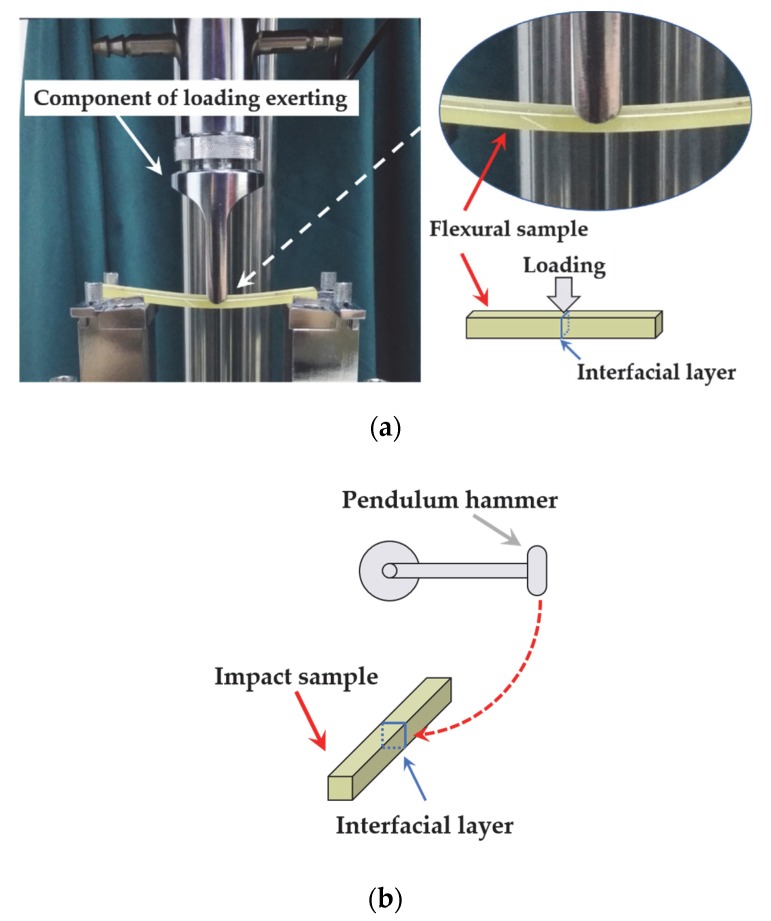
Photo of the arrangement in flexural test and impact test of the sample with laminated interface: (**a**) the test of flexural strength; (**b**) the test of impact strength. The load was applied on the interfacial region during the test of flexural strength and impact strength.

The tensile tests were conducted using the SANS ZMGI 250 machine with a speed of 2 mm/min according to ASTM D-638. Flexural tests were also conducted using the SANS ZMGI 250 machine fitted with a three-point bending fixture, and the bending speed was 2 mm/min according to ASTM D-790. The SANS ZBC1251 Charpy tester was used to test the impact strength of unnotched samples according to ASTM D-256. The test span for flexural and impact tests was 70 mm. All results were averaged over 10 samples. All mechanical tests mentioned above were performed at room temperature (about 26 °C). For samples with interfacial structures, the experimental layout for the flexural test and impact test was as shown in Figure 1.

**Figure 2:**
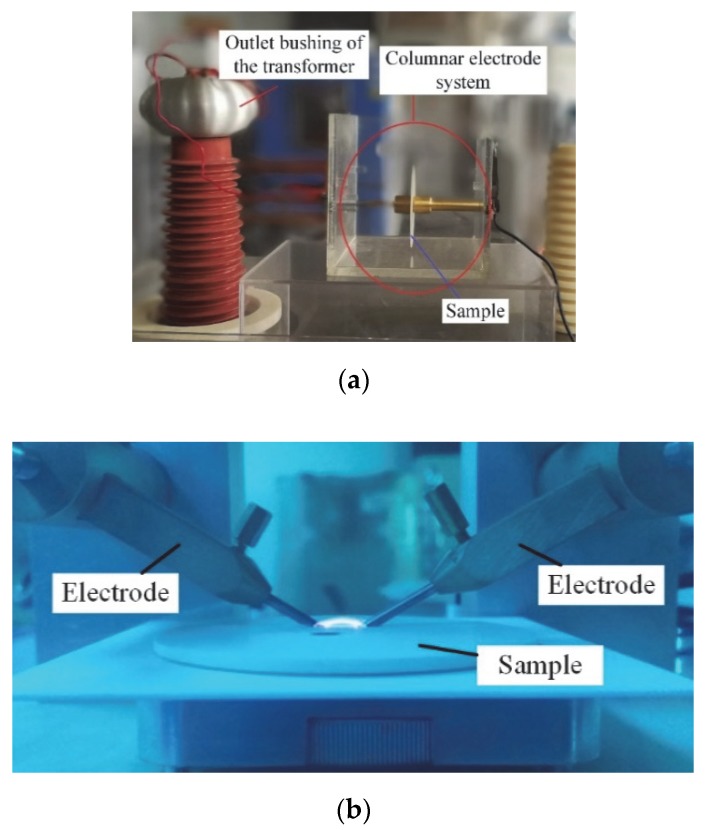
Photo of the breakdown system and the arrangement in arc ablation resistance test: (**a**) breakdown system; (**b**) arrangement in arc ablation resistance test.

The lab-tailored breakdown test system mainly consisted of transformers and electrodes as shown in Figure 2a. The electrodes and the sample were immersed in transformer oil to prevent flashover along the surface. The diameter of the electrodes was 25 mm, and the sample size was *Φ* 100 mm × 1 mm. The samples with an interface had a double-layered structure, with a thickness of 0.5mm for each layer. One electrode was loaded with an increasing AC voltage at the rate of 1 kV/s, while the other was grounded. The breakdown strength for both kinds of samples (with and without interface) were averaged over 10 tests. All breakdown tests were performed at about 25 °C.

The arc ablation resistance tests were conducted using the Huace HCDH-20 KV. The arc ablation resistance can be used to evaluate the surface flashover performance of dielectric materials. Two separated electrodes were mounted on a flat sample. The arc could be generated between the two electrodes by applying a high voltage and low current. The sample was gradually destroyed by the arc ablation, thus resulting in a conductive path formed on the sample. The corresponding experimental set-up is shown in Figure 2b.

The diameter of the electrode was about 2.4 mm, and the end of the electrode in contact with the specimen was sharpened as required. The distance between the tips of both electrodes was 6.35 mm. The applied current on the electrode as a function of time followed a periodic pattern as shown in Table 1. The arc strength increased discontinuously with time. The thickness of all samples was more than 3 mm. As before, all results were averaged over 10 tests.

### 2.3. Sample Preparation

#### 2.3.1. Curing Process of Epoxy Resin

The preparation and curing processes of epoxy resin samples without an interface were as follows: firstly, the epoxy resin, curing agent, and accelerator were mixed evenly at room temperature in the weight proportion of 100:65.5:0.5 and then poured into the molds. Secondly, the molds containing the mixture were placed into the vacuum oven for degassing and then cured for 2 h at 80 °C, 4 h at 120 °C, and 4 h at 150 °C. Finally, the samples were gradually cooled down and then successively taken out from the oven and mold.

**Figure 3:**
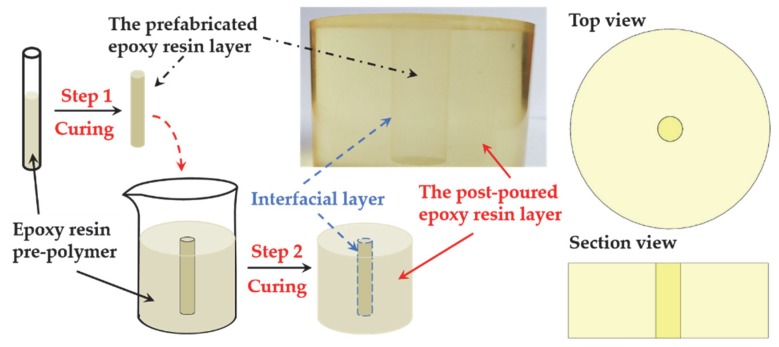
Schematic diagram of the preparation process of sample with an interfacial layer. For step 1, the epoxy resin pre-polymer was poured into a test tube, and the prefabricated epoxy resin layer (marked with a black dot-dashed line) was obtained after curing. For step 2, the prefabricated layer was placed into a mold, and the same pre-polymer in step 1 was poured into the beaker. Finally, after curing, the sample was obtained with an interfacial layer (marked with a blue dashed line) between the prefabricated layer and the post-pouring layer (marked with a red solid line).

The preparation process of the laminated epoxy sample with interface was as follows: the same mixture was poured into the molds with half occupation (with a thickness of 0.5 mm in the double-layered sample) and then degassed and cured in the oven following the same procedures stated above; when the samples were cooled down to room temperature, the rest of the mixture was poured into the mold to occupy the other half of the space in the mold, and then the degassing and curing processes were successively repeated. Finally, the double-layered epoxy resin samples with an interlaminar interface were, thus, obtained. The corresponding procedures are illustrated in Figure 3. However, in order to display the interlaminar interface more intuitively, the shape of the sample prepared in Figure 3 was not the sample used for measurement.

For the sample prepared in Figure 3, the two layers of epoxy resins were of the same formula. The only difference was that one layer was the prefabricated epoxy resin and the other was the post-curing epoxy resin. A slight boundary could be observed at the interface between two layers. As the refractive index at the interface changed due to the existence of the interlaminar interface, the two layers of epoxy resin could be distinguished.

#### 2.3.2. Preparation of Sample for Mechanical Test

**Figure 4:**
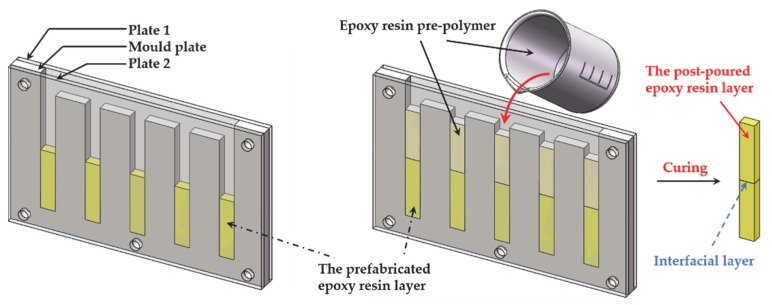
The preparation process of the mechanical samples.

The tests of tensile strength, bending strength, and impact strength were employed to measure the bonding strength at the interface. These mechanical tests were performed by using the samples and molds. The preparation process of samples used in impact test is shown in Figure 4, and the preparation process of the other two kinds of mechanical samples was similar. The prepared half samples were put into the mold and then another pouring and curing process was carried out to form the sample with an interface.

#### 2.3.3. Preparation of Sample for Dielectric Measurement

**Figure 5:**
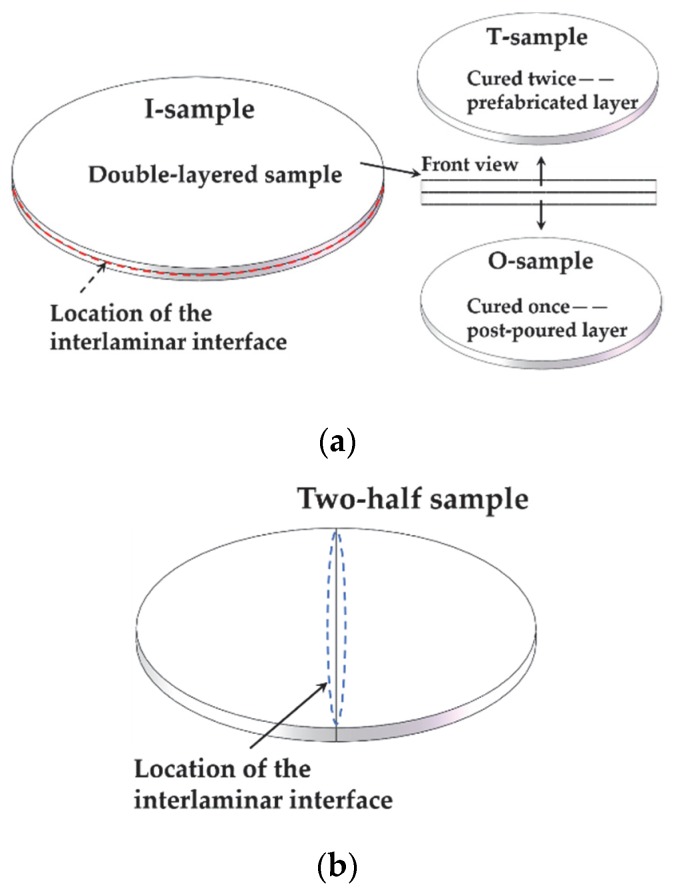
Schematic diagrams of interface position in the interface samples: (**a**) different types of samples; (**b**) two-half sample.

In order to investigate the dielectric properties of the interface between the two layers and the layers themselves, three types of samples were prepared: double-layered laminated epoxy samples with an interface for studying the interfacial influences on relevant properties; prefabricated single-layered epoxy samples prepared via two successive curing processes for studying bulk properties of the top layer in laminated two-layered epoxy samples; post-curing single-layered epoxy samples prepared via one curing process for studying the bulk properties of the bottom layer in laminated two-layered epoxy samples. The schematic diagrams of these three types of samples are illustrated in Figure 5a, and their characteristics are summarized in Table 2.

To allow simple comparison and analysis, the prefabricated single-layered epoxy sample was named the T-sample, the post-curing single-layered epoxy sample was named the O-sample, and the double-layered laminated epoxy sample was named the I-sample.

In the measurement of arc ablation resistance, the interfacial samples could be divided into two types. One was the double-layered sample as shown in Figure 5a, and the other was the two-half sample as shown in Figure 5b. The positions of the interfaces were different in these two types of samples, but the interfaces were both formed via two successive curing processes for each layer/part.

## 3. Results and Discussion

### 3.1. Microstructure Analysis of the Interlaminar Interface

**Figure 6:**
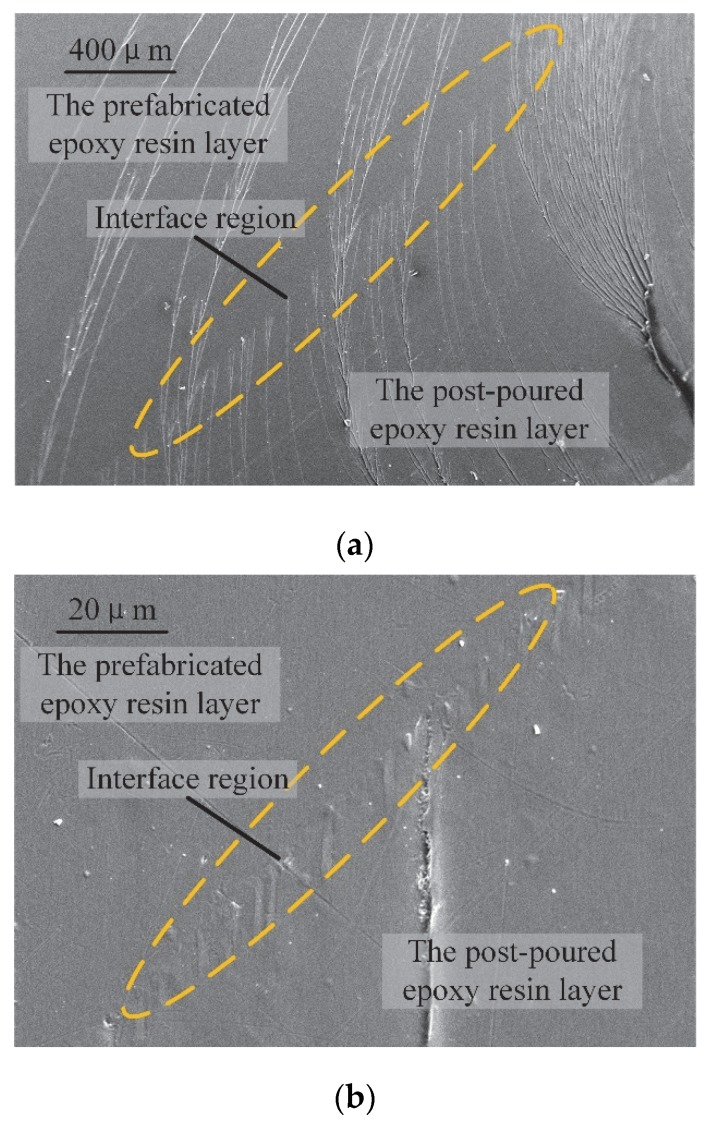
Fracture microstructure of interface sample: (**a**) 50× magnification; (**b**) 1000× magnification.

In order to study the morphological interfacial properties of multi-layered epoxy resin, the interfacial microstructure was studied. The fracture microstructures of the I-sample with different magnifications observed by SEM are shown in Figure 6.

No obvious boundary could be found near the interface, but the interface itself could be located by distinguishing it from the different development of cracks on different layers. As shown within the dashed eclipse region in Figure 6, some cracks crossed the interface while others terminated at the interface. This phenomenon of crack development near the interface shows that the interface somehow hindered the crack growth. When the cracks approach the interface, the dissipation of fracture energy may stop some cracks from growing, resulting in the termination of some cracks at the interface. However, no defects could be observed in the region where the cracks terminated.

Folds appeared at the interface of the post-curing epoxy resin layer, and the length of the folds was less than 10 μm. These folds may have been caused by the mismatch in volume contractions of the two adjacent epoxy resin layers near the interface during the curing process. During the curing process of the post-curing epoxy resin layer in double-layered samples, the prefabricated epoxy resin layer was already cured once before; thus, its volume contraction was only due to the cooling of temperatures. On the other hand, for the post-curing epoxy resin layer, the volume contraction was not only caused by cooling but also by the process of curing itself. This mismatch of volume contractions occurred during the curing process when the temperature was relatively higher. At this temperature, the post-curing epoxy resin layer was not yet fully cured. The stress could be absorbed through deformation, and the folds formed in consequence.

According to the microscopic morphology analysis at the interface, we found that the two adjacent epoxy layers near the interface were closely bonded, and no bubbles or defects could be observed at the interface. Therefore, for manufacturing multi-layered epoxy resin, obvious defects can be avoided near the interface if the preparation process is properly controlled.

### 3.2. Mechanical Strength

**Figure 7:**
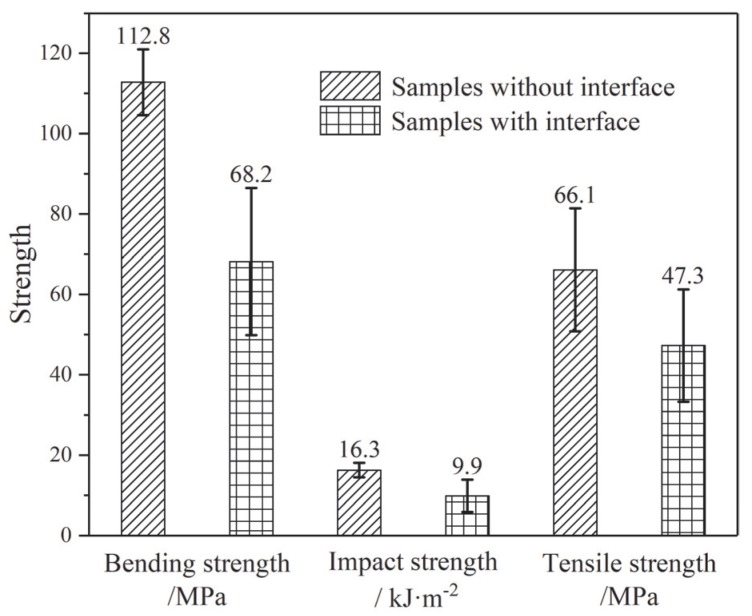
Mechanical strength of samples with or without interfaces.

The mechanical strength of multi-layered epoxy graded material is directly affected by the bonding strength between adjacent layers near the interlaminar interface. Tensile strength, bending strength, and impact strength are important indexes to characterize the mechanical properties of epoxy resin castings. Therefore, the bonding strength at the interface was studied using the mechanical strength test mentioned above. The test results of the samples with and without the interface are shown in Figure 7.

The existence of an interface reduced the three kinds of mechanical strength. For I-samples, the tensile strength, bending strength, and impact strength were reduced by 28.4%, 39.5%, and 39.2% respectively, compared to those for single-layered samples without interfaces. All I-samples were destroyed along the region of the interlaminar interface, indicating that the interface was the weak point of the multi-layered epoxy when referring to mechanical strength.

The mechanical failure of the samples without an interlaminar interface was mainly caused by the breaking of chemical bonds in the bulk due to external forces. Therefore, the fracture strength mainly characterized the total bonding energy along the crack. Although the mechanical strength was decreased in presence of the interface, the reductions of the three mechanical strengths were less than 40%. This level of reduction indicated the formation of covalent bonds between the two layers of epoxy resins near the interface. If no covalent bond was formed near interface, the mechanical strength of double-layered samples with the presence of the interface would have been reduced more tremendously. This is mainly because the energy of intermolecular forces is more than an order of magnitude smaller than that of covalent bonds [28].

For the prefabricated epoxy resin layer, there were more incompletely cured groups on its surface than in its bulk after the curing process. Due to the limited thickness of the surface layer, the proportion of the incompletely cured groups was small; thus, the effect of these incompletely cross-linked groups on the properties of the material could be neglected [28]. During the fabrication process of I-samples, the uncured epoxy resin contacted the surface of the prefabricated epoxy resin layer, which was already cured. Therefore, the functional groups in the uncured epoxy resin could move and react with the functional groups which were incompletely reacted in the prefabricated epoxy resin layer, resulting in possible covalent-bonded crosslinking at the interface. However, due to the limited movement of groups on the surface of the prefabricated epoxy resin layer, the probability of a cross-linking reaction at the interface was lower than that inside the bulk. That is to say, a “weak cross-linking” reaction may occur at the interface, which would be the main reason for the reduction in mechanical strength.

The covalent bonds formed at interface were covalent bonds related to the cross-linking process. These types of covalent bonds were similar to those in the bulk of cured epoxy layers, but the cross-linking probability at the interface was lower than that in bulk. Meanwhile, due to the thinness of the interface layer, it was difficult to characterize its micro-structure, which could only be postulated from its macro-properties. Therefore, the research approaches and the characterizations of this interface need to be studied extensively.

For laminated epoxy dielectric graded materials, the mechanical strength decreased in the presence of the interface by less than 40%, which is not fatal. Therefore, this material can still act as mechanical support, but shear stress between layers should be avoided as much as possible.

### 3.3. Dielectric Strength

Dielectric strength is an important research index for dielectric graded materials [29]. In this study, the dielectric strength was mainly characterized by breakdown strength and arc ablation resistance. These two kinds of strength were measured and analyzed for epoxy samples with and without interface structure.

#### 3.3.1. AC Breakdown Strength

Breakdown strength is a comprehensive dielectric property of insulating materials. The experimental data of breakdown strength are generally processed using a Weibull distribution function.

**Figure 8:**
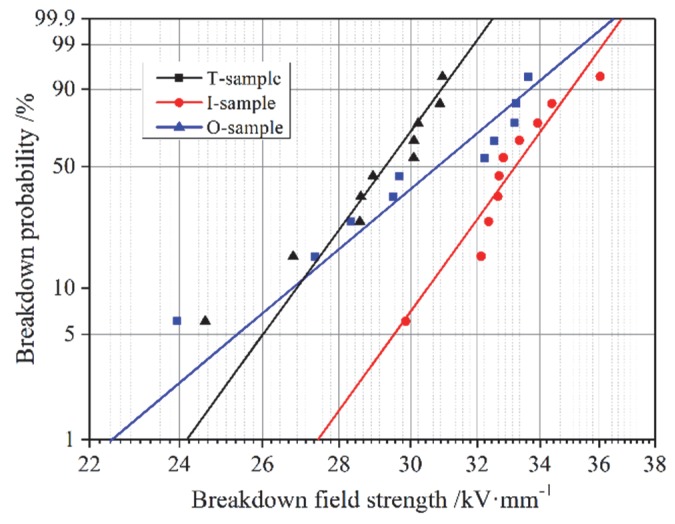
Weibull distribution curves of breakdown field strength of samples.

The Weibull distribution model is based on weakness theory. The whole system is regarded as a series of subsystems; thus, the strength of the system depends on the strength of the weakest link [30]. The two-parameter Weibull distribution function is widely used for analyzing experimental data of the breakdown strength, and the function is shown in Equation (1).

(1) $$F\left(x\right)=1-\exp\left(-\frac{x^{\beta }}{\alpha }\right),$$

where *x* is the breakdown field strength and its unit is/kV·mm^−1^, *α* is the scale parameter and its unit is/kV·mm^−1^, and *β* is a shape parameter, which determines the shape of the density function. It is a slope parameter in the two-parameter Weibull distribution. The breakdown field strengths of the three kinds of samples were measured. The results are shown in Figure 8, and the scale and shape parameters are shown in Table 3.

Due to the existence of the interface, the breakdown field strength increased from 31.64 kV/mm to 33.75 kV/mm, and the lowest breakdown field strength was obtained by the T-sample. The breakdown field strength of the I-sample was strongest among the three types of samples.

The breakdown process of the material is the result of the carriers’ movement under the electric field. The interface region can affect the carriers’ behavior in two ways: interfacial polarization and trapping [27]. The heterogeneous permittivity and conductivity across the interface between the materials in adjacent layers are the main factors causing the interface polarization. However, the epoxy resin layers on both sides of the interface in our study were of the same formulation. Therefore, the interfacial polarization had little contribution to the migration of the carriers. However, the folds observed by SEM in the I-sample near the interface, as well as the “weak cross-linking” at the interface, occurred during the second curing process, generating physical and chemical defects, thereby resulting in lot of deep traps created at the interlaminar interface [31]. The carrier’s directional migration process under the electric field is a process of its trapping and detrapping. The introduction of deep traps by the interface makes it difficult for the trapped carriers to move away, as more energy is required for the trapped carrier to overcome the potential well for detrapping. As a result, the interface in the I-sample hinders the movement of carriers along the direction of the electrical field. Moreover, according to References [21,23], the interface between solid insulating layers mainly accumulates negative charges in a DC field. Under an AC field, the traps produced by “weak cross-linking” can also capture negative carriers.

**Figure 9:**
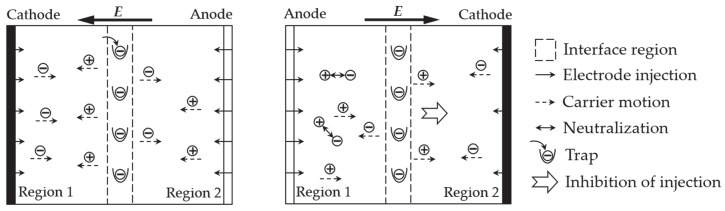
Schematic diagram of the effect of interface region on carrier motion in a cycle of alternating current (AC) voltage.

Moreover, the main reason accounting for AC breakdown is due to the accumulation of charges from the vicinity of the electrodes [32,33,34]. A schematic diagram of the carrier motion in the I-sample under a cycle of AC voltage is demonstrated in Figure 9.

As shown in the left panel of Figure 9, the electrons are trapped at the interface before passing through the interface under the first half-cycle of AC voltage. With the reversal of electric field polarity in the second half-cycle of AC voltage, the polarity of trapped interface charges remains the same as that of the right electrode, as shown in the right panel of Figure 9. The accumulation of the homopolar charge (negative) between the interface and cathode reduces the local electric field in region 2 and, thus, inhibits the injection of negative charges from cathode. Similarly, the local electric field in region 1 should be slightly enhanced due to the accumulation of heteropolar charge between the anode and interface. Nevertheless, negative carriers travel faster than positive carriers; thus, negative carriers are more likely to gather near the anode, thereby neutralizing the positive carriers injected from the electrode, as shown in region 1 in the right panel of Figure 9. As a result, injection of the positive carriers is reduced in the second half-cycle of AC voltage. In conclusion, during the whole cycle of AC voltage, the injections of both positive and negative carriers from both electrodes are reduced in the presence of an interlaminar interface. In other words, the existence of an interface in double-layered laminated epoxy inhibits the carrier’s injection from electrodes. The trapped charge is equivalent to an interfacial barrier.

The analysis in the paragraph above is in accordance with Reference [32]. This phenomenon inhibits the charge injection, thus enhancing the AC breakdown voltage. However, the increment is not significant due to the small proportion of the interface region in the double-layered sample. Therefore, the AC breakdown strength of the I-sample is slightly higher than that of the O-sample.

The shape parameter reflects the dispersion of data. Shape parameters have a negative correlation with data dispersion. The shape parameter of I-samples is the largest, indicating a better homogeneity of the material, which is possibly due to the effect of deep traps introduced by the interface on carriers counteracting the influences of some defects.

#### 3.3.2. Arc Ablation Resistance

The arc ablation resistance test is a comprehensive property of interfacial insulating material, involving thermal properties, electrical properties, and the microstructure of materials. In addition, surface flashover is an important dielectric performance of insulating materials. In the process of surface flashover, arc develops along the dielectric surface [35]. Thus, the strength of arc ablation resistance can also be used to characterize the ablation during the surface flashover.

An arc forms between the electrodes after the test begins. At the beginning, the arc is in the air on top of the surface of the sample. As the degree of carbonization caused by ablation intensifies, the arc gradually moves into the bulk of the sample until a conducting carbonization channel is formed. Meanwhile, the arc disappears completely on the sample surface. A longer arc ablation time results in a larger ablation resistance of the sample.

**Figure 10:**
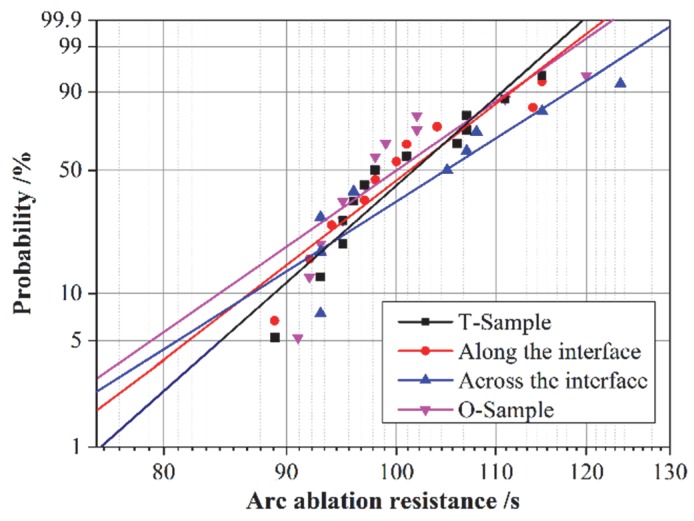
Weibull distribution of the arc ablation resistance of the different samples.

In order to study the interface influences on arc ablation resistance, the two-half samples with an interface in between two half-circled epoxy plates were used, as shown in Figure 5b. The arc ablation resistance at the interface was measured via two methods. In one method, the two electrodes were placed along surface of the interface in order to make sure that the entire arc was generated alongside the direction of the surface crack of the interface. In the other method, the electrodes were placed on each counterpart of the interface in order for the arc to occur across the interface. The arc ablation resistance test results of I-samples using these two methods were analyzed and then compared with the corresponding results of the other two types of samples without an interface. The experimental results are plotted and fit by a Weibull distribution in Figure 10. The scale parameter *α* and the shape parameter *β* are listed in Table 4.

The ablation results along the interface path were close to those of the samples without interlaminar interfaces. The arc ablation resistance across the interface was the biggest among all. The arc resistance time increased from 103.4 s to 108.7 s when the arc crossed the interface.

During the arc resistance experiment, the interlaminar interface can capture carriers. The voltage applied during the experiment was 50 Hz AC. Therefore, as stated in Section 3.3.1, the charge accumulated at interface hindered the carrier injection from the electrode. As a result, the number of injected carriers reaching the counter side of the electrode decreased, leading to the enhancement of arc ablation resistance. In contrast, the arc ablation resistance for cases where arc ablation occurred along the interface did not change much. There were two factors affecting the ablation resistance under this circumstance. On one hand, the effect of trapping carriers still existed. On the other hand, the lower degree of cross-linking at interface led to faster decomposition while arcing. Therefore, the effects of these two factors counteracted each other, leading to a negligible change in arc ablation resistance.

The shape parameters *β* were similar in all four situations. In the case of the arc crossing the interface, the shape parameters *β* were the smallest. This was mainly because the contact width between the arc and the interface was the width of the arc across the interface, which was rather small. Therefore, the shielding effect of the charge accumulated at the interface was not that obvious.

The results of the breakdown test and arc ablation resistance test in accordance with each other showed that both properties for the double-layered samples were slightly enhanced in a similar range. The existence of an interface increased these two aspects of dielectric strength to varying degrees. The main reason accounting for this effect is that the introduction of traps by the interface would capture carriers, thus hindering the carrier migration. As a consequence, we can conclude that the effect of the interlaminar interface on dielectric strength is trivial if the adjacent epoxy layers near the interface are of the same formula. However, it should also be noted that, if the material parameters on both sides of the interface are significantly different, the interface polarization behavior should be carefully considered.

## 4. Conclusions

In this paper, the influence of interlaminar interface on the mechanical and dielectric strength of the laminated epoxy graded dielectric materials was studied based on double-layered laminated epoxy samples.

During the preparation of double-layered laminated epoxy samples, a “weak cross-linking” reaction may occur at the interface between the two adjacent layers. This weak covalent bonding at the interface leads to a reduction of the mechanical strength with respect to the bulk of the epoxy resin, but it is still stronger than that derived from intermolecular forces. As a result, the decrease in mechanical strength of the double-layered sample with an interface is less than 40%, which is good enough to meet mechanical requirements for the application of power equipment.

The interface region may exhibit the capability of capturing negative carriers. The presence of an interface in double-layered laminated epoxy inhibits the carrier’s injection from electrodes, leading to a slight enhancement of the AC breakdown field strength and ablation resistance of samples with interfaces. However, this improvement is not significant due to the small proportion of the interface region in the double-layered sample.

In conclusion, the interface structure has a certain effect on the mechanical and electrical strength of the multi-layered material. If a more delicate interface treatment method can be developed, multi-layered epoxy insulation with better mechanical and dielectric properties can be achieved and applied in a much broader range of power equipment.

## Figures and Tables

**Table 1:** Parameters of each stage in arc ablation resistance test.

Stage	Current (mA)	Cycle Time (s)	Total Time (s)
1/8	10	1/8 on, 7/8 off	60
1/4	10	1/4 on, 3/4 off	120

**Table 2:** Characteristics of the different types of samples.

Sample Type	Sample Characteristics	Sample Name
Prefabricated layer	Samples underwent two-step curing processes	T-sample
Samples with interface	Samples with an interlaminar interface	I-sample
Post-curing layer	Sample underwent one-step curing process	O-sample

**Table 3:** Weibull distribution parameters of breakdown field strength of samples.

Sample Type	Scale Parameter *α* (kV·mm^−1^)	Shape Parameter *β*
T-sample	29.76	22.06
I-sample	33.75	22.25
O-sample	31.64	13.46

**Table 4:** Weibull distribution parameters of arc ablation resistance of different samples.

Sample Type	Scale Parameter *α* (kV·mm^−1^)	Shape Parameter *β*
T-sample	104.3	14.1
Along the interface	104.3	12.3
Cross the interface	108.7	10.1
O-sample	103.4	11.1
